# Traditional and Medical Applications of Fasting

**DOI:** 10.3390/nu14030433

**Published:** 2022-01-19

**Authors:** Francesco Visioli, Carla Mucignat-Caretta, Francesca Anile, Stefan-Alexandru Panaite

**Affiliations:** 1 Department of Molecular Medicine, University of Padova, Viale G. Colombo, 335121 Padova, Italy; carla.mucignat@unipd.it (C.M.-C.); francesca.anile@studenti.unipd.it (F.A.); 2 IMDEA-Food, 28049 Madrid, Spain; 3 Department of Cardiac, Thoracic, Vascular Sciences, and Public Health, School of Hygiene and Preventive Medicine, University of Padova, 35121 Padova, Italy; stefanalexandru.panaite@studenti.unipd.it

**Keywords:** fasting, health-span, longevity, chemoprevention, evolution

## Abstract

Fasting has been practiced for millennia, for religious, ethical, or health reasons. It is also commonplace among different species, from humans, to animals, to lower eukaryotes. Research on fasting is gaining traction based on recent studies that show its role in many adaptive cellular responses such as the reduction of oxidative damage and inflammation, increase of energy metabolism, and in boosting cellular protection. In this expert review, we recount the historical evolution of fasting and we critically analyze its current medical applications, including benefits and caveats. Based on the available data, we conclude that the manipulation of dietary intake, in the form of calorie restriction, intermittent fasting, dietary restriction with the exclusion of some nutrients, prolonged fasting, and so forth, is anthropologically engraved in human culture possibly because of its positive health effects. Indeed, many studies show that fasting ameliorates many biochemical parameters related to cardiovascular and cancer risk, and neurodegeneration. Mechanistic studies are plentiful, but largely limited to cell cultures or laboratory animals. Understandably, there are no controlled trials of any form of fasting that gauge the effects on [any cause] mortality. Physicians should be aware that misinformation is pervasive and that their patients often adopt dietary regimens that are far from being clinically validated. Moreover, doctors are often unaware of their patients’ religious or traditional fasting and of its potential health effects. Based on current evidence, no long-term fasting should be undertaken without medical supervision until future research will hopefully help shed further light on fasting and its effects on human health.

## 1. Introduction

### 1.1. What Is Fasting

Fasting has been practiced for millennia, for religious, ethical, or health reasons. It is also commonplace among different species, from humans, to animals to lower eukaryotes [1]. Research on fasting is gaining traction based on recent studies that show its role in many adaptive cellular responses such as reduction of oxidative damage and inflammation, increase of energy metabolism, and in boosting cellular protection [2].

Fasting is the word that describes a period of voluntary abstinence from food and drink, usually longer than the physiological overnight fast [3]. It is different from calorie restriction (CR), in which the daily caloric intake is decreased by 20–40% for a long period of time [1].

Fasting can be conveniently subdivided into three categories, depending on the time schedule: alternate-day fasting, time-restricted feeding, and whole-day fasts (Table 1). Alternate-day fasting involves the interchange between ad libitum feeding and fasting days, consisting of 0% to 25% of daily caloric needs intake. Time-restricted feeding follows the same eating routine every day: a certain number of hours (16–20) of fasting and the remaining hours (4–6) to feed. Whole-day fasting consists of one to two days of complete fasting per week, interchanged with ad libitum eating on the other days [4].

In the latest years, all different fasting protocols are being investigated as a strategy for weight loss and purported health benefits. In fact, fasting leads to great metabolic changes: the main change is known as glucose-to-ketone switch (G-to-K), meaning that with lack of feeding, ketones and fatty acids are the major cellular fuel for brain and for the whole body. G-to-K switch also has metabolic effects: it decreases blood sugar, insulin levels as well as IGF-1 levels, stimulates glycogen release and increases lipolysis and ketogenesis [5,6]. Furthermore, fasting plays a role in the adaptive cellular response; in the reduction of oxidative damage and inflammation; in cellular protection; and in autophagy [7].

To understand the molecular mechanisms of fasting, many models have been studied: in lower eukaryotes, chronic fasting raises longevity, because of a reorganization in metabolic and stress resistance pathways; in rats, intermittent or periodic fasting protects against cancer, diabetes, heart disease, and neurodegeneration; in humans, it reduces the consequences of, for example, obesity, hypertension, asthma, and rheumatoid arthritis [2].

In this narrative review, we recount the historical evolution of fasting and we critically analyze its current medical applications, including benefits and caveats.

### 1.2. Practice of Fasting in the Monotheist Religions

#### 1.2.1. Judaism

In Judaism there are six days of fasting throughout the year. The most important fast is Yom Kippur, on the 10th of Tishrei, which is currently considered in Judaism the day of judgment and the holy day of the year. The source of fasting on Yom Kippur is a commandment in the Torah, and its purpose is not mourning, but the torture of the body for the purpose of transcendence and purification of the soul.

Four more fasts were determined following the destruction of the First Temple and the Second Temple, and they mark mourning for the destruction: (1) Tisha B’Av—the main day of fasting in memory of the destruction, and it marks the day when Jerusalem and the two temples were destroyed. This is also the day when the desert generation is doomed not to enter the land following the sin of the spies; (2) Seventeen of Tammuz—marks the day the walls of Jerusalem were breached by Titus’ army, before the destruction of the Second Temple; (3) Gedaliah’s fast, on Tuesday, Tishrei—marks the day that Gedaliah son of Ahikam, the Jewish commissioner who remained in the Jerusalem area after the first destruction, was murdered. This is the first known political assassination in the history of the Jewish people; (4) Ten Tevet—the day of the beginning of Nebuchadnezzar’s siege of Jerusalem, at the end of which Jerusalem was conquered and destroyed.

Apart from these fasts, Judaism marks another day of fasting—the fast of Esther on the 3rd of Adar, the day before Purim. There is another fast that applies only to the firstborn called Ta’anit Bechorot on the 4th of Nisan.

On Yom Kippur and Tisha B’Av the fast lasts a full day, from the previous day until the evening of the next day. The rest of the fasts are lighter and begin only in the morning of that day. Another difference is that in light fasts only eating and drinking are forbidden, compared to Yom Kippur and Tisha B’Av where there are four additional prohibitions: washing, lubricating, bedding (having sex), and locking leather shoes.

As with Ramadan (see below), abstention from food is meant to improve one’s ability to focus on repentance and on spiritual elevation. Jewish belief holds that on Rosh Hashanah, God makes judgment for each one, and on Yom Kippur, “judgement is sealed” [8].

The peculiarity of Yom Kippur lays in its short, that is, 25 h, duration. While period is too short to bring about untoward health effects, it must be underscored that orthodox Jews also abstain from water intake, which might lead to dehydration, especially in hot climates. Immediate consequences include headache (particularly in heavy coffee drinkers who have to go cold turkey) and dizziness [9]. However, there is no evidence of serious side effects of Yom Kippur, as suggested by the unchanged rates of emergency room admissions [10].

#### 1.2.2. Christianity

As humans are body and—for the believers—soul, it would be useless to imagine a purely spiritual religion: in order to engage, the soul needs the acts and attitudes of the body. Fasting, always accompanied by a suppliant prayer, serves to express humility before God: fasting (Lev 16:31) is equivalent to “humiliating one’s soul” (Lev 16:29). Fasting is therefore not an ascetic feat; it does not aim at procuring some state of psychological or religious exaltation. Similar uses are attested in the history of religions. In the biblical context, when humans abstain from eating for a whole day (Judg 20:26; 2 Sam 12:16 f; Jon 3:7) while considering food as a gift from God (Deut 8:3), this deprivation is a religious act whose reasons must be understood exactly; the same applies to abstention from marital relations. According to the scriptures, worshippers turn to the Lord (Dan 9:3; Eze 8:21) in an attitude of dependence and total abandonment: before facing a difficult task (Judg 20:26; Est 4:16), or again to beg forgiveness of a fault (1 Kings 21:27), to solicit a healing (2 Sam 12:6–22), while complaining on the occasion of a burial (1 Sam 31:13; 2 Sam 1:2), after a widowhood (Jdt 8:5; Lk 2:27) or following a national misfortune (1 Sam 7:6; 2 Sam 1:12; Bar 1:5; Zec 8:19), to obtain the cessation of a calamity (Joel 2:12–17; Jdt 4:9–13), to open oneself to divine light (Dan 10:12), to await the grace necessary for the fulfillment of a mission (Acts 13:2 s), to prepare oneself for an encounter with God (Ex 34:28; Dan 9:3). The occasions and reasons are varied, but, in all cases, it is a matter of placing oneself in God’s presence with faith, in an attitude of humility to welcome God’s action. This profound intention reveals the meaning of the forty days spent without food by Moses (Ex 34:28) and by Elijah (1 Kings 19:8). As for the forty days of Jesus in the desert, which are modeled on this double example, they do not have the purpose of opening him to the Spirit of God, because he is already full of it (Lk 4:1); if the Spirit impels him to this fast, he does so to inaugurate his messianic mission with an act of trusting abandonment in his Father (Mt 4:14).

If Jesus does not prescribe anything of the kind to his disciples (Mk 2:18), it is not because he despises this justice or wants to abolish it, but because he comes to apply it; therefore, he forbids showing it off and invites, on some points, to overcome it (Mt 5:17–20; 6:1). Jesus insists more on detachment from riches (Mt 19:21), on voluntary continence (Mt 19:12) and—above all—on self-denial to carry the Cross (Mt 10:38–39). In fact, the practice of fasting is not exempt from certain risks: danger of formalism, already denounced by the prophets (Am 5:21; Jer 14:12); danger of pride and ostentation, if people fast “to be seen by men” (Mt 6:16). To please God, true fasting must be combined with love of neighbor and imply a search for true justice (Is 58:2–11); it cannot be separated from either almsgiving or prayer. Finally, according to the scriptures, people must fast for the love of God (Zec 7:5). Jesus, therefore, invites persons to do it with perfect discretion: known by God alone, this fast will be the pure expression of hope in him, a humble fast that will open the heart to interior justice, the work of the Father who sees and acts in secret (Mt 6:17 ff). In matters of fasting the Church preserved the customs of Judaism, carried out in the spirit defined by Jesus. The Acts of the Apostles mention cultural celebrations involving fasting and prayer (Acts 13:2 ff; 14:23). During his extended apostolic work, Paul is not satisfied with suffering hunger and thirst when circumstances demand it; he adds repeated fasts to it (2 Cor 6:5; 11:27). The Church has remained faithful to this tradition, seeking—with the practice of fasting—to put the believers in an attitude of total openness to the grace of the Lord, awaiting his return. In fact, if the first coming of Christ put an end to the expectation of Israel, the time that follows from his resurrection is not that of total joy in which the acts of penance would be out of place.

Defending, against the Pharisees, his disciples who did not fast, Jesus himself said: “Can the friends of the bridegroom fast as long as the bridegroom is with them? The days will come when the bridegroom will be taken away from them, and then in those days they will fast” (Mk 2:19). While waiting for the bridegroom to return to us, penitential fasting has its place in the practices of the Church.

#### 1.2.3. Islam

Fasting (*as-saum*) during the month of Ramadhan (which falls on the ninth month of Islamic Lunar Hijri Calendar) is one of the main teachings, that is, one of the five pillars of Islam [11,12]. The Holy Quran states “O you who believe, fasting is prescribed for you as it was prescribed for those who preceded you. Perhaps you will become fearful” (Surat ul-Baqarah, 2: 183). According to this verse, the tradition of fasting is not exclusively Islamic; for Christians and Jews it is a tradition as described above. Muslims must fast during the month of Ramadhan, meaning that all Muslims who are in normal physical condition and are adults must totally and completely abstain from eating, drinking, smoking, and having sexual intercourse. This abstinence must be realized every day from sunrise to sunset. The duration of the days during the month of Ramadhan depends on the season in which this month is located. Since the Islamic calendar is based on the lunar system, it is ten days shorter than the solar calendar, hence its location changes every year.

“Eat and drink until, at dawn, you can distinguish the ‘white thread’ [very early morning] from the ‘black thread’ [the darkness of the night], then fast until night falls” (Surat ul-Baqarah, 2: 187). Thus, during the month of Ramadhan, the usual hours of a Muslim family change, giving rise to a form of intermittent fasting. Fasting must be broken, as the Quran clearly indicates, “*ilal layl*”, that is, after sunset. Elderly people, the infirm, travelers, pregnant women and nursing mothers are exempt from fasting in the month of Ramadhan. Some of these people, such as the infirm, pregnant women or nursing mothers, have to make up for the days they have missed when their situation returns to normal. The elderly, as it will be difficult for them to fast, will be exempt but will have to pay a certain amount of money to the poor as a ransom.

According to Islam, there are various reasons and benefits associated with the fasting tradition. Some are spiritual and some are social in nature. As regards the spiritual dimension, fasting during the month of Ramadhan is not for atonement or repentance. It is not some kind of punishment; it is a religious rite for a positive purpose. The main goal is “so you will become fearful [of Allah]!”.

Approximating other Asian traditions, according to Islam it is only through control—not total suppression—of anger and desire that a person can achieve a balanced personality. Islam demands that its followers control their lives and not become slaves to their desires.

Therefore, by the end of the month, the Muslim hopes to have become a spiritually stronger person who controls his own life. There are also other days in the Islamic calendar on which fasting is recommended but not compulsory. These include the first and last Thursday of each month, Mondays, 13th, 14th and 15th day of each month.

The month of Ramadhan also has a particular social dimension. When Muslims fast during the days of this month, they are hungry and thirsty; during this time of hunger and thirst, they can better understand the suffering of hungry and poor people in the community around the world. Knowing that people around the world have no access to food or water stimulates the feeling of charity and compassion in practitioners. In this sense, the month of Ramadhan is also a month of compassion and charity. At the end of the fasting month, Muslims celebrate “Eid ul-Fitr” and gather in mosques or in open places for prayers and special celebrations.

Although Ramadan cannot be seen as a prolonged fasting, but, rather, an intermittent form of fasting, its metabolic effects have been reported by several investigations [13], which considerably contribute to our understanding of this self-prescribed practice. Indeed, given that there are approximately 1.8 billion Muslims in the world, Ramadan is likely the largest worldwide ongoing cohort trial on intermittent fasting [14]. In this context, and pending appropriate trials, Ramadan is likely to be beneficial for healthy individuals. However, it is important to underscore that some patients, for example, diabetics [15,16] should be carefully evaluated by their physicians before undertaking Ramadan. Even though studies on the safety of Ramadan fasting in patients with cardiac disease are scant and there are no randomized trials, some consensus has been reached [17]. The ‘low-moderate risk’ group may fast, provided their medications and clinical conditions allow. The ‘high’ or ‘very high risk’ groups should not fast and may consider safe alternatives such as non-consecutive fasts or fasting shorter days, for example, during winter. Indeed, even Ṣalāḥ al-Dīn Yūsuf ibn Ayyūb (Saladin), one of the most famous Muslim heroes, died with high fever while trying to make up for two months of missed fasting [18].

In summary, Ramadan is a form of intermittent fasting that takes place once a year and might bring about positive metabolic effects in the majority of healthy people. Any kind of disease requires careful supervision by general practitioners or specialists [14,19] (Table 2).

### 1.3. Other Religions

#### 1.3.1. Buddhism

According to several reports, for example, the Buddha Carita by Aśvaghoṣa, the Shakyamuni Buddha underwent several periods of extreme fasting (up to, according to popular wisdom, eating only one grain of rice per day) as one of the ascetic means to reach enlightenment. These austerities did not lead to spiritual progress but did cause him to become emaciated as shown by many statues found in East Asia. In the end, Buddha criticized the fasting practiced by Indian ascetics of his day, for example, the Jains (see below), and focused, instead, on meditation and mindful eating.

Nowadays, most Buddhist schools recommend that monks do not eat after noon. In addition, several periods of prolonged, that is, several days fasts carried out during the year are part of many Buddhist traditions, for example, *danjiki* in Japanese Tendai, *geumchok* in Korean Seon, or *zhai* in China.

It is also worth mentioning the extreme practice of self-mummification (*Sokushinbutsu* in Japanese) via starvation. Some such mummies are found in the Himalayan region and in some mountainous areas of Japan [20].

#### 1.3.2. Jainism

Jainism uses several types of fasting to purify both the body and the soul, in addition to performing a penance. Most Jains fast at special times such as birthdays, anniversaries, during festivals, and on holy days, but the most interesting form of Jainism fasting is the *sallekhanā* or *santhara*, that is, a ritual slow “suicide” in which the intake of food and fluids is gradually reduced [21]. This is traditionally done under the guidance of a spiritual guide and is seen as an instrument of spirituality and when inevitability of death is a matter of undisputed certainty [21]. Santhara is being challenged by human rights activists, but proponents rebut that Santhara cannot be termed “suicide” and is, in fact, an act of faith, rational thinking, and courage.

### 1.4. Modern Fasting Protocols: The Buchinger-Wilhelmi Example

Modern fasting protocols were born with the German Dr. Otto Buchinger, who treated and solved his infected joint rheumatism by using unconventional medicinal approaches such as fasting. The success of this form of treatment led him, his daughter Maria Buchinger, and his son-in-law Helmut Wilhelmi, to set up a fasting clinic in Germany. Later, a second clinic was opened in Spain and both are still operating, promoting scientific research along with therapeutic fasting (www.buchinger-wilhelmi.com/en/, accessed on 16 November 2021). According to his own guidelines, the Buchinger’s fasting method is based on a daily intake of fruit or vegetable juice (¼ Lt), vegetable broth (¼ Lt), honey (30 g) and 2–2.5 Lts of fluids such as herbal teas and water. In addition, during the fasting periods, a combination of physiotherapy, mind-body methods, psychotherapy as well as physical activity are added to the program [22]. The effectiveness of a medically-supervised, therapeutic fasting protocol is suggested by some published data, but the most recent work consists of a one-year observational study with 1422 subjects, participating in a fasting program based on fasting periods between 4 and 21 days. Subjects selected one out of four established programs of 5, 10, 15, and 20 fasting days, and they were asked to drink 3 L/day of water or non-caloric teas with an optional addition of 20 g of honey. Moreover, they had organic fruit or vegetable juice (250 mL) for lunch and, for dinner, a vegetable soup (250 mL); the total calorie intake was 200–250 kcal per day. Participation in the study was voluntary and involved people 18 to 99 years old whose main personal goals were to decrease cardiovascular risk factors, weight loss in case of obesity, and amelioration of general health problems such as inflammatory diseases, tension, and fatigue. The parameters measured in the study were: weight, abdominal circumference, blood pressure, pulse, ketone bodies, blood composition (in terms of blood sugar concentration, lipid parameters and blood count), in addition to self-reported well-being and potential adverse effects. The results show that the fasting protocol led to marked weight loss. Furthermore, overweight, abdominal circumference, blood pressure, and other cardiovascular risk factors, improved. Some altered blood parameters were also normalized and major health complaints in most participants improved [5].

Other studies, later discussed, suggest potential benefits for cardiovascular diseases, obesity, diabetes, and cancer treatment, indicating fasting under controlled regimen as a potential medical tool.

### 1.5. Prolonged Fasting in Humans

Strong human evidence of any health effect induced by prolonged fasting is scant. The first report of a protracted fast is likely that of FG Benedict [23], who published the blood biochemistry, urine analyses, and vital signs of a Maltese man of unknown age, who voluntarily fasted for 31 days. Of note, the intestinal microbiota (back then still called “flora”) was analyzed by unknown methods [23]. The subject was able to exist in a fairly normal mental condition, entirely out of proportion to the physical decline in the body functions. Anticipating findings from WWII (see below), the subject broke the fast by ingesting excessive amounts of fruit juices, which caused gastro-intestinal distress and required hospitalization [23]. The subject lost 21.9% of his bodyweight [24], which is in keeping with what reported by Korbonits et al. after the David Blaine stunt (see below) [25,26] and with previous and subsequent reports [24,27].

In terms of the longest-lasting fast, the record goes to Scottish man Angus Barbieri, who fasted for 382 days, from June 1965 to July 1966, during which he lost 125 Kg (initial weight: 207 Kg) [28]. During the 382 days of his fast, vitamin supplements were given daily as ‘Multivite’ (BDH), vitamin C and yeast for the first 10 months and as ‘Paladac’, for the last 3 months. Non-caloric fluids were allowed ad libitum. From Day 93 to Day 162 only, he was given potassium supplements (two effervescent potassium tablets BPC supplying 13 mEq daily) and from Day 345 to Day 355 only he was given sodium supplements (2.5 g sodium chloride daily) [28]. Refeeding was done slowly, via administration of small amounts of carbohydrates [28] and a boiled egg [29]. The patient died at 51 [29].

As mentioned, sound data on the health effects of prolonged fasts are scant. Thomson et al. [30] published their experience with 13 obese patients hospitalized in Glasgow, U.K. who were treated with non-caloric fluid-only fasts—supplemented with vitamins—for 25–249 days. The authors conclude that total fasts are well-tolerated and that the patient’s attitude and motivation are paramount to successfully complete the therapeutic regimens [30]. However, the authors also warn that this therapy should only be carried out under close medical supervision owing to the possibility of electrolyte imbalance or other untoward effects.

### 1.6. Refeeding

Refeeding individuals who endured a prolonged fast should be done with extreme caution [31,32,33]. Indeed, according to some estimates, at least 500 POWs from the Auschwitz concentration camp and as many as 14,000 from the Belsen concentration camp died of refeeding syndrome (https://westcorkpeople.ie/columnists/the-history-corner-columnists/when-food-can-kill-a-lesson-from-wwii/, accessed on 16 November 2021). Edema, acute heart failure and acute vitamin B1 deficiency are likely responsible for the observed rapid death of refed starved individuals.

Opportunities to study the effects of prolonged fasting and subsequent refeeding in healthy individuals are rare. The first well-documented study of refeeding under controlled conditions was that of performance artist David Blaine who, in 2005, ingested only water during a 44-day fast in London, UK [26]. During this stunt, he lost 25% of his body weight [25]. Upon arrival at a hospital, Blaine exhibited normal plasma glucose, normal cholesterol, and triacylglycerol, but considerably elevated free fatty acids and 3-hydroxybutyrate [25]. During refeeding, biochemical analyses revealed vitamin deficiencies, abnormal liver function, low IGF-I, leptin, and low ghrelin levels, as well as a transient increase in plasma orexin and resistin levels at the time of increase in subjective feeling of hunger [25]. Of note, upon admission, that is, after 44-day water fast, the subject had a body mass index of 21, which would not alert any physician to the risk of starting an uncontrolled dietary regimen [25]. In summary, the only controlled refeeding study we are aware of calls for caution when individuals resume eating after prolonged fasting for purported health purposes. Electrolyte imbalance is likely to occur and any extreme diet (including fasting) should be carefully discussed with the general practitioner.

### 1.7. Fasting and Animals

All animals require food, which provides the fuel and supportive compounds for all of the body’s activities, from the routine cellular processes to movement, growth, and reproduction. Nevertheless, animals in nature rarely experience continuous intake of food: animals eat and digest following their rhythmic circadian pattern [34]. If food becomes limited, feeding is stopped or reduced. As an example, phocid seal pups are nursed almost ceaselessly for days, but within a few months they are abandoned on the ice or onshore without food when their mothers return to the sea [35]. Indeed, fasting cycles occur to many organisms, which can explain behavioral, morphological, metabolic and physiological adaptations needed to reduce metabolic expenditure and preserve homeostasis [36]. In animal models, many fasting habits are observed and vary depending on the species, on the environment, and on the physiological needs. The observation of animal fasting patterns is useful to -at least partially- elucidate human physiology when in this state.

Fasting habits can be conveniently listed as follows: (a) Hibernation: a seasonal state of dormancy to face harsh environmental conditions, for example, low temperatures and food scarcity; (b) Estivation: a seasonal state of dormancy to face harsh environmental conditions, for example, high temperatures and water scarcity; (c) Migration: changing environment to reach the food source; (d) Natural, that is, lack of food by chance [37].

When animals undergo fasting, some metabolic pathways change and a downregulation of tissue function and structure is generally observed. In fact, cellular metabolism is reduced and the rate of substrate catabolism and tissue degradation is decreased. The concurrent switch from carbohydrate to lipid metabolism stems from the near depletion of glycogen stores and a consequent reliance on the catabolism of energy-dense lipid stores. Fasting in animals can begin when a defined quantity of fuel is available, which determines the duration an individual can survive without food. Of course, basal metabolism, thermoregulation, physical activity, and reproduction must be considered parts of the metabolic expenditure and differ among different species. Usually, metabolic costs reduction is the most used strategy for extended periods of fasting; however, this strategy cannot be used if the animal is engaged in energy-consuming activities such as migration, incubation of eggs, feeding, mating, or territory defense. It is necessary to avoid dynamic actions to downregulate the metabolic pathways necessary to survive during fasting. Actually, in fasting conditions, glycogen, fats, and proteins are no longer synthesized, thus promoting the downregulation of metabolic rates [38].

During fasting, animals experience daily torpor that, actively or passively, lowers body temperature, contributing to slacken metabolic processes, as well as cardiac and ventilatory activities [39]. Although body temperature plays a role in metabolic pathways, the downregulation of cellular processes can also depress metabolic rate by a further 30% to 85%. Mechanisms involved concern the suppression of RNA and protein synthesis, the depression of ion pumps, and the reduction in mitochondrial proton leak [40].

As discussed before, when food is not available, animals use endogenous lipids, glucose, and amino acids to produce the ATP necessary to maintain vital activities and cellular processes.

#### 1.7.1. Glucose and Glycogen

Glucose is indispensable to many organs, especially for the functions of the central nervous system, the renal medulla, and for the production of mature erythrocytes. In addition to dietary intake, glucose availability is guaranteed by de novo synthesis from amino acids, glycerol, ketones, and recycled lactate and pyruvate in the liver and kidney [41,42]. Glucose can also be obtained by using glycogen stored in liver and skeletal muscles—when blood glucose decreases, glycogen is cleaved to glucose-6-phosphate, rapidly metabolized in tissues or converted in the liver into glucose.

#### 1.7.2. Lipids

Lipids are stored as pads of fat in body cavity such as mesenteric fat, as intracellular droplets within the tissues like muscle and liver and subcutaneously. Lipids are found in triglycerides, fat bodies and droplets originating from ingested lipids when energy intake exceeds energy expenditure. Before predicted fasting periods, animals usually increase the intake of lipids to build up stores of fat in their body; during fasting there is a metabolic switch from carbohydrates to lipids, used as an energy source. Nevertheless, especially for hibernating mammals, a change in gene expression is observed: genes regulating carbohydrate metabolism decrease in favor of an increase of genes regulating lipid metabolism [43]. Triglycerides are also transformed into non-esterified fatty acids (NEFAs) and glycerol, a source of available energy for animals. Concerning NEFAs, their beta-oxidation generates acetyl-CoA, which is shuttled to TCA cycle to produce ketone bodies. Ketones can also be used as an alternative metabolic substrate to glucose for muscles, kidneys and -most important- for the central nervous system [44].

#### 1.7.3. Proteins and Amino Acids

In animals undergoing fasting, proteins are catabolized into amino acids, which can take part in gluconeogenesis, the process involved in the production of glucose. During long periods of low-calorie intake, especially in animals that fast during the entire winter, protein catabolism is regulated by shifts in expression of genes related to protein utilization [44]. Most of the amino acids come from the breakdown of tissue proteins, which can be dangerous if fasting is prolonged. In fact, fasting can induce a continuous degradation of structural proteins, resulting in the deterioration of critical tissues and organs and in a severe homeostatic imbalance. However, the most serious consequence of protein depletion, such as cachexia and death, are observed when 50% of proteins pool is consumed [36]. In addition, amino acid catabolism results in the production of ammonia, a toxic compound, that needs to be quickly removed or detoxified by the conversion to urea or uric acid.

### 1.8. Fasting and Human Health: The Available Data

As discussed above, fasting has been practiced since a long time, for many different reasons: religious, ethical, or for health. Protocols of fasting roughly follow three modalities: whole-day fasts, time-restricted feeding, and alternate-day fasting, better known as intermittent fasting. Nowadays, intermittent fasting (IF) is the most popular and touted way to lose weight and increase lifespan [1]. However, IF has other noticeable effects: in addition to reducing body weight, it affects glucose regulation, reduces systemic inflammation, and ameliorates some cardiometabolic parameters. IF can be difficult to integrate in a daily routine, chiefly because of the social norms and conventions about eating. On the other hand, fasting is the simplest form of dieting, convenient in terms of saving money and saving mental energy, usually used to think what to eat and when to eat [45].

In addition to diet trends and opinions, fasting has attracted the interest of medical research since 1945, when University of Chicago scientists reported that alternate-day feeding extended the life span of rats [46]. Recently, IF has been studied in several pilot studies, has been compared to calorie restriction and most importantly has been declared as a prevention factor for many diseases, regarding metabolic issues, heart disease, brain illnesses and cancer [2].

#### 1.8.1. Fasting and Brain Function

Our current understanding of the impact of fasting on cognitive functions and on the nervous system is largely provided by animal studies of IF. In fact, severe food deprivation in mammals results in a decrease of the size of most organs, save for the brain and the testicles. In rodents, alternating ad-libitum days and IF can enhance brain function, learning and memory as much as improvements in performance on motor and sensory function tests [47]. The behavioral responses to intermittent fasting seem to associate with increased synaptic plasticity and enhanced production of new neurons from neural stem cells [48].

A particular and specific role, regarding the adaptive responses of the brain to food scarcity during human evolution, is played by the brain-derived growth factor (BDNF). In fact, BDNF is involved in the regulation of energy intake/expenditure balance, owing to the fact that both BDNF and insulin receptors are coupled to the PI3-kinase AKT and MAP kinase signaling pathways [2].

Fasting may increase neuronal activity in brain regions involved in cognition, resulting in larger production of BDNF, enhanced synaptic plasticity and improved stress tolerance [49]. Moreover, BDNF signaling in the brain may also mediate metabolic responses to fasting such as regulation of appetite, activity levels, peripheral glucose metabolism, and autonomic control of the cardiovascular and gastrointestinal systems [50].

Interestingly, animals on an IF diet also recover better after acute injury, including severe epileptic seizures, stroke, and traumatic brain and spinal cord injuries when compared to ad libitum-fed animals [51,52].

Unfortunately, data on the effects of IF on human brain are inconsistent and inherently difficult to perform, but some studies show an improvement on visual memory and on cerebrospinal fluid biomarkers of brain bioenergetics in subjects with mild cognitive impairment, after one month on a low glycemic diet [53].

#### 1.8.2. Fasting and Obesity

Overweight and obesity are defined as abnormal or excessive fat accumulation that presents a risk to health. A body mass index (BMI) over 25 is considered overweight, and over 30 is obese; over 4 million people die each year for being overweight or obese. Obesity has a multifactorial etiology, yet it is mainly caused by an imbalance between calories consumed and expended. In recent decades, there has been an increase in the consumption of energy-dense foods, high in fat and free sugars and a decrease in physical activity due to the changing nature of many types of work, more access to transportation and increased urbanization.

To lower the risk of overweight and obesity, calorie intake must be reduced, by increasing the portion of daily intake of fruit, vegetables, legumes, whole grains, and nuts, while concomitantly practicing physical activity (60 min per day for children and 150 min per week for adults; https://www.who.int/health-topics/obesity#tab=tab_3, accessed on 16 November 2021).

Due to high recidivism rate in calorie restriction diets, an alternative method of reducing energy intake can be fasting [54]. According to short-term IF studies, weight can be reduced by 3% to 8% over 2 to 12 weeks in obese or overweight adults [55]. Unfortunately, these studies were lacking a control group or a comparison between IF and no-intervention control [56]. A pilot study including these variables was conducted at the University of Colorado Anschutz Medical Campus. The 26 enrolled volunteers were 18–55 years old, had a BMI ≥ 30 kg/m^2^, and were non-smokers. Furthermore, they were excluded if presenting diseases such as diabetes, cardiovascular diseases, hypertension, cancer, thyroid disease, seizures, migraines, significant renal, hepatic, or gastrointestinal disorders, binge eating disorder, current depression, history of bariatric surgery, or were taking medications known to affect appetite or energy metabolism [57]. The aim of the study was to evaluate the safety and tolerability of an 8-week zero-calorie alternate day fast (ADF) intervention, followed by 24 weeks of unsupervised follow-up to assess risk of regaining weight after the trial. Of 26 participants, only 21 completed the 24-week unsupervised follow-up. Secondary aims were to prove efficacy of IF compared with moderate daily CR in generating changes in weight, body composition, lipids, and insulin sensitivity index. At the end of the 8-week intervention, absolute weight change (CR −7.1 ± 1.0 kg, IF −8.2 ± 0.9 kg) did not differ between groups but there was a marginally significant between-group difference in relative weight change (CR −6.2 ± 0.9%, IF −8.8 ± 0.9%). On the other hand, the composition of weight-regain tended to differ between groups: CR gained 1.2 ± 0.8 kg of fat mass and 1.1 ± 0.5 kg of lean mass, and IF lost 20.4 ± 0.8 kg of fat mass and gained 2.0 ± 0.5 kg of lean mass. At week 8, total cholesterol, HDL, and LDL decreased significantly in both groups and triglycerides decreased significantly in IF, but there were no differences between the two groups. On the contrary, fasting glucose and triglycerides decreased significantly in IF at 8 weeks; however, there were no between-group differences in change in metabolic parameters, suggesting the diets had similar effects on these metabolic disease risk indicators. These results are similar to those of prior studies, which suggest that IF produces improvements in fasting lipids [58,59].

In summary, from the available research it appears that IF programs are able to reduce body weight (3–7% on average), body fat (3–5.5 kg on average), total cholesterol (10–21%), and triglycerides (14–42%) in normal-weight, overweight, and obese humans [4]. In any case, food restriction for overweight must be conducted under medical supervision to avoid the risks of diets that restrict energy too severely, such as semistarvation and very low-calorie diets. Semistarvation can lead to hyperphagic responses and increases in fat mass beyond initial levels and a very low-calorie diet can endanger physical and mental health [60].

#### 1.8.3. Fasting and Hypertension

Hypertension is a serious medical condition and can increase the risk of several diseases. It is a major cause of premature death worldwide, with upwards of one in four men and one in five women—over a billion people—having the condition. Reducing modifiable risk factors is the best way to prevent hypertension and associated diseases; these factors include physical inactivity, consumption of tobacco and alcohol, being overweight or obese and unhealthy diets (https://www.who.int/health-topics/hypertension, accessed on 16 November 2021). Although anti-hypertensive medications are extensively used, dietary and lifestyle modifications are also effective in the treatment of patients with hypertension. In fact, drugs may have a positive benefit for certain patients, but most people who die of coronary artery disease, congestive heart failure, or stroke do not have blood pressure (BP) values in sufficiently elevated ranges to warrant drug treatment.

A study run by the NHS, National Health Association, and the International Association of Hygienic Physicians, enrolled 174 adult patients suffering from essential hypertension and other health problems, over a period of 12 years. Volunteers included in the trial presented systolic BP of at least 140 mmHg, diastolic BP of at least 90 mmHg or both, and underwent a period of water-only fasting ranging from 4 to 28 days. Before starting the program, patients were instructed to eat a diet consisting exclusively of fresh raw fruits and vegetables and steamed vegetables for 2 days. Once the transition diet program and examination procedures were completed, patients began the water-only fasting program. Furthermore, every participant, along with the water-fasting program, followed its own therapeutic plan, but BP medications used at entry were gradually phased down. With the beginning of fasting, daily activities also decreased because even moderate activity during a water-only fast can double energy use. After the fasting period, a supervised refeeding started with the consumption of juices made from fruits and vegetables at the first stage, followed by a diet including fresh fruits and vegetables, steamed and baked vegetables, whole grains and legumes, and small quantities of raw unsalted nuts and seeds as stage 2. The diet specifically excluded any meat, fish, fowl, eggs, dairy products, or added oil, salt, or sugar. Bread products and other processed foods were also excluded.

Participants’ results were later divided into four groups, describing four relevant time points: (1) baseline; (2) start of water-only fasting; (3) end of water-only fasting; and (4) end of supervised refeeding. The effects of water-only fasting and refeeding on BP were statistically significant. In fact, BP dropped during the prefasting, water-only fasting, and post-fasting periods. A greater decrease in BP occurred during the water-only fasting period and the overall BP mean drop was of 37.1/13.3 mmHg.

Moreover, the drop in BP led 6.3% of the patients taking antihypertensive drugs during the study to suspend the use of these drugs during the water-only fast and the supervised refeeding periods. Body weight over the entire treatment period decreased by an average of 6.9 kg. These results document the effectiveness of water-only fasting and dietary restriction for the treatment of patients with hypertension, in fact nearly nine (89%) of ten patients who suffered from hypertension at entry were normotensive by the end of their supervised refeeding period. The results also suggest that fasting is relatively safe because no morbidities were observed at any point in the study. Interestingly, hunger was not reported as a problem after the second or third day of water-only fasting. On the other hand, twenty (11%) patients, although exhibiting substantial decreases in BP, remained hypertensive at the conclusion of treatment. It is important to note that these partial responders had a significantly higher average systolic BP at entry. This finding suggests the need of further studies to understand how water-only fasting affects blood pressure and to study the long-term effectiveness of this strategy and the potential adverse effects.

For now, water-only fasting remains a potentially useful therapeutic strategy that must be experienced under medical supervision but cannot replace drug therapy [61].

#### 1.8.4. Fasting and Cancer

Cancer is the second leading cause of death globally, accounting for an estimated 9.6 million deaths, or one in six deaths, in 2020 [62]. Lung, prostate, colorectal, stomach and liver cancer are the most common types of cancer in men, while breast, colorectal, lung, cervical and thyroid cancer are the most common among women [62]. Early diagnosis and treatment of cancer is as important as prevention: between 30% and 50% of cancer deaths could be prevented by modifying or avoiding risk factors [63]. Additionally, prevention offers the most cost-effective long-term strategy for the control of cancer. Risk factors are many and nowadays well known, such as the use of tobacco or excessive alcohol intake, but also diet and food intake plays a key role in the prevention program [63]: the WHO recommends maintaining a healthy weight and eating a healthy diet with fruit and vegetables as a way to prevent cancer (https://www.who.int/health-topics/cancer#tab=tab_3, accessed on 16 November 2021).

In mice, alternate-day fasting leads to a great reduction in the incidence of lymphomas [64] and fasting for 1 day per week retards spontaneous tumorigenesis in p53-deficient mice [65]. In fact, if fasting is applied correctly, that is, without malnutrition, it might have cancer-preventive effects in the presence of carcinogens: multiple cycles of periodic fasting showed the same efficacy as chemotherapy in the treatment of some cancers in mice [66]. On the other hand, fasting has been suggested to have positive effects on cancer treatment. Intermittent fasting for 2–3 days appears to protect mice from a variety of chemotherapy drugs’ untoward effects. The mechanism of this effect is called differential stress resistance and is responsible for protection of normal cells, but not cancer cells during short-term starvation [67].

Also, the combination of fasting and chemotherapy fosters an extreme environment for cancer cells: they are unable to adapt to extreme conditions. This phenomenon is called differential stress sensitization (DSS) and occurs because most of the mutations are deleterious for cancer cells, and once accumulated they promote growth under standard conditions and sensitize them to extreme environments [68]. In metastatic tumors in mice, combinations of fasting and chemotherapy that cause DSR and DSS result in 20–60% cancer-free survival compared to chemotherapy or fasting alone, which are generally not sufficient to cause any cancer-free survival [68]. For humans, randomized clinical trials are not yet available, but preclinical and clinical research suggest the potential application in prevention and treatment of human cancer. In fact, IF protects normal cells/tissues from chemotherapy-induced toxicity [66] while simultaneously increasing its therapeutic efficacy on a wide variety of malignant cells [69].

In a pilot study, ten patients diagnosed with a variety of malignancies voluntarily fasted for up to 180 h before and after chemotherapy treatment (48–140 h before and 5–56 h after). Patients received an average of four cycles of various chemotherapy drugs in combination with fasting, with encouraging results: although fasting did not prevent the chemotherapy-induced reduction of tumor volume or tumor markers, they reported fasting to decrease significant side effects such as vomiting, diarrhea, fatigue and weakness. On the contrary, the same side effects were experimented from patients undergoing chemotherapy accompanied with a regular diet [70].

As mentioned, the use of fasting (namely, IF) in oncology is gaining attention and is becoming very popular among patients, even in the current absence of conclusive data. As recently reviewed by Gabel et al. [71], available data show that IF may hold potential to improve effectiveness of chemotherapy, decrease treatment related side effects, while ameliorating treatment related decreases in quality of life and daily functioning It is imperative that further studies are conducted to better understand the potential role of fasting, combined with chemotherapy cycles, on tumor regression. For now, the reduction of chemotherapy’s side effects induced by IF should not be underestimated and is worth appropriate large-scale clinical trials.

### 1.9. Mechanisms of Action

To the best of our knowledge, the precise mechanisms of action of prolonged fasting have never been thoroughly investigated [72]. However, there is much information on less radical forms of fasting, in particular calorie restriction (CR). CR is one of the (if not the) most-studied anti-aging interventions, especially in animals, with research going back over 100 years. Even though a detailed analysis of CR is beyond the scope of this review and some discrepancies have been reported, some shared results have been published and are worth discussing to better understand the mechanisms of action of food restriction.

The longevity-promoting effects of moderate starvation have been known as far back as 1914 [73], when a study showed that caloric restriction in mice inhibited tumor growth. In 1935 another study showed that caloric restriction increased average rat lifespan from 483 days to 894 days [74]. The human equivalent of this effect would theoretically be huge reaching seven decades of added lifespan. Perhaps most interesting, animal, namely rodents’ studies of calorie restriction constantly show that animals that are fed approximately 30% fewer calories often die without any apparent sign of diseases. Some authors term this phenomenon “compression of morbidity” [75,76,77]. From an epistemological viewpoint this represents an important paradigmatic shift from the quixotic search of lifespan extension that characterized human history (see for example the fountain of youth that led Juan Ponce de Leon to discover Florida [78]) to the currently more socially relevant aim at increasing health span.

The definition of calorie restriction is that of a reduction of dietary intake below energy requirements while maintaining optimal nutrition. In short, energy intake reduction without malnutrition. CR is different from fasting, but both are included in dietary restriction (DR) models [79]. In fact, DR involves modification not just in calorie intake, but also in dietary composition or timing of food intake (such as intermittent fasting, IF) and is being studied for the prevention or treatment of chronic diseases [80]. Furthermore, recent studies show how CR and some forms of IF promote stress resistance and longevity in model organisms, from unicellular yeast to mammals, suggesting that many of the molecular protective mechanisms of CR have probably evolved billions of years ago and are partially shared by many species [2].

As a caveat, it must be underscored that CR in laboratory animals unwillingly induces prolonged day fasts because animals rapidly, that is, ~2 h, consume their entire daily meal then fast until the next day [81]. Thus, CR data from laboratory animals must be interpreted with extreme caution as they might be the consequence of collaterally imposed fast [82]. An additional caveat concerns the use of in vitro models, which provide useful mechanistic information, but are quite remote from in vivo ones; for example, cell cultures are nearly devoid of antioxidants [83,84] and essential fatty acids [85].

In yeasts, flies, fish and rodents, CR extends lifespan, and the level of dietary restriction necessary to see such effects ranges between 10 to 50% compared to fed ad libitum mammals. The extension of lifespan due to CR has been studied in several works and involves many metabolic pathways [34].

Mechanistically, calorie restriction reduces the activity of various signal transduction pathways either directly (yeast) or indirectly through the reduced levels of growth factors such as IGF-1 (worms, flies, mammals). The role of TOR and S6K in promoting aging appears to be conserved in yeast, worms, flies, and mice. By contrast, the AC-PKA pathways and the TOR- S6K pathway promote aging in yeast and mammals, whereas an insulin/IGF-1– like receptor or the upstream growth hormone (mammals) accelerates aging in worms, flies, and mice. Similar transcription factors (GIS1, MSN2/4, DAF-16, FOXO) inactivated by either the AC/PKA, IGF-1/AKT, or TOR/S6K pathways affect cellular protection and/or aging in all the major model organisms. Notably, in the multicellular worms, flies, and mice, these genes may promote aging within the cells in which they are expressed but also in other cells through the regulation of circulating factors. The mechanisms proposed for the longevity extension caused by inhibition of these nutrient signaling pathways include a decrease in the free radical superoxide (mediated in part by SODs) and of its damage to macromolecules, protection of proteins by chaperones (Hsp70), decreased translation, the activation of autophagy, and the switch to hypoxia-associated gene expression patterns (in yeast and mice). In yeast, the effects of DR on life- span extension are also associated with reduced activities of the Tor-Sch9 and Ras-AC-PKA pathways and require the serine-threonine kinase Rim15 and transcription factors Gis1, Msn2, and Msn4. In worms, transcription factors regulated by the TOR-S6K and AGE-1-AKT pathways are implicated in the anti- aging effects of DR and, in flies, reduced activity of both Ins/IGF-1 and TOR can protect against shortening of life span by increased food intake, although in both worms and flies deletion of DAF-2/FOXO shortens life span, but the animal continues to respond to DR. In mice the longevity effects of DR appear to involve reduced activity of the GHR/IGF-1 pathway because DR does not extend further the life span of GHR-deficient mice.

Whether—and to what extent—the aforementioned biochemical pathways are affected in humans by fasting remains to be determined. However, a very recent and important publication by Pak et al. [82] demonstrate that daily prolonged fasting, and not solely reduced caloric intake, is likely responsible for the metabolic and geroprotective benefits of a CR diet.

#### Ketone Bodies as Potential Effectors of Fasting

As mentioned above, fasting stimulates a G-to-K switch, thereby increasing the circulating concentrations of ketone bodies (KB) [86,87]. The biological effects of enhanced ketosis are still not fully elucidated. On the one hand, KB have been suggested to reduce oxidative stress, associated with endogenous histone deacetylase inhibition [88]. However, other mechanisms of action have been proposed. In particular, oxidative stress induced by KD metabolism might be beneficial in the long term because it initiates an adaptive (hormetic) response characterized by the activation of the master regulators of cell-protective mechanism, nuclear factor erythroid 2-related factor 2 (Nrf2), sirtuins, and AMP-activated kinase [89]. Enhanced autophagy induced by KB is also likely to explain the potential beneficial effects of fasting [89,90].

In terms of redox status and its modulation to enhance longevity, the emerging role of vitagenes is the subject of much investigation [91]. In short, a preconditioning signal leading to cellular protection through hormesis [92] is an important redox dependent aging-associated to free radicals’ species accumulation and inflammatory responses involved in neurodegenerative/neuroprotective mechanisms [93]. Several phytochemicals are being investigated to elucidate their role in mitochondrial protection [94,95,96] and it is conceivable to hypothesize a similar mechanism of action of KB.

To prove the purported beneficial effects of fasting-induced KB production, the best way is to perform clinical trials with exogenous KB [97]. Although data are still scant, there is some preliminary evidence of the positive effects of KB administration in some physio-pathological conditions. One recent example is that of mild cognitive impairment, of which an alteration of brain phospholipid’s profile is a hallmark [98], opening avenues for therapeutic interventions [99,100]. In a recent randomized trial, the use of ketogenic medium-chain triglycerides improved cognitive outcomes in mild cognitive impairment subjects [101,102], confirming in a “pharmacological” setting accumulated data on the beneficial effects of ketosis in, for example, seizure management [103].

In summary, fasting and associated ketosis (when not taken to extreme levels) are likely to promote hormetic-like effects in various organs such as the brain and the skeletal muscle, in turn preventing for example, mitochondrial decay, promote autophagy and mitochondriogenesis, and improve overall health.

## 2. Conclusions and Future Directions

Fasting of any kind is an inextricable feature of human evolution and, for whatever reason, appears to be engraved in human behavior. For the near totality of our history, food was not readily available and humans are hardwired to endure short or prolonged periods with no intake of calories. Unfortunately, this still happens in several countries, where food security is a major issue [104]. For the first time, though, the percentage of people who are overweight and overfed surpassed that of people who have limited access to proper meals [105]. This leads to health deficiencies, skyrocketing global obesity rates, excess chronic diseases, and premature mortality. One solution that is being proposed is to adopt some type of calorie restriction, for example, alternate-day fasting (which 10% of the American population already does [106]). Indeed, accrued data strongly suggest that short periods of fasting or CR without malnutrition might lessen the noxious effects of inflammation [107] and possibly prevent cardiovascular disease [108] and cancer [109]. An active role in cancer therapy has also been proposed [110], but it is very important to highlight that cancer cells are able to use many energy substrates, in a Darwinian manner: as a result, cancer patients often lose weight and this loss could progress to sarcopenia and cachexia [111], calling for extreme caution when fasting protocols are applied in oncology.

The overarching goal of fasting is to increase lifespan and, more importantly, healthy life span. Concerning the former, a debate [112,113] based on limited data is taking place on whether there is a limit to the human lifespan [114], which some authors controversially [115] place at around 115 years [114] and others attribute to progressive loss of resilience [116].

From a medical viewpoint, it is important to underscore that data on the effects of fasting on health and longevity are scant and that long-term trials that include hard endpoints such as all-cause mortality have never been performed and very likely never will be.

Physicians should be aware that many of their patients already adopt some type of fasting, including prolonged ones with the aim to “detoxify” their body. This is especially commonplace in oncological patients who undergo chemotherapy [117,118], very often without telling their physicians. Anecdotal reports of two- or three-day water fasts before chemotherapy [119] underline the need for the medical community to gain control over this practice and carefully discuss the pros and cons with their patients. As a pertinent example, extreme diets, for example, those that include fasts could lead to orthorexic behaviors [120] and other eating disorders.

Alternatively, patients should be aware that, despite historical practices, the overall and long-term health and longevity benefits of fasting are not well understood at this time. For this reason, such personal practices should always be divulged to healthcare professionals in the clinical setting to improve holistic understanding of the health situation and therapeutic progress.

In this paper, we provide a comprehensive historical survey of fasting traditions as well as relevant clinical findings. It is hoped that this effort will draw attention to the need for cellular and clinical research regarding possible benefits of the fasting practice in health patients as well as those under physician’s care. In this modern age of metabolic disease such as obesity and its medical consequences, forensic study of long-held practices such as fasting will likely lead to better understanding of these societal problems as well as improved therapeutic and behavioral practices.

In conclusion, the manipulation of dietary intake, in the form of calorie restriction, intermittent fasting, dietary restriction with exclusion of some nutrients, prolonged fasting and so forth, is anthropologically engraved in human culture possibly because of its positive health effects. Indeed, many studies now show that fasting ameliorates many biochemical parameters related to cardiovascular and cancer risk, and neurodegeneration. Mechanistic studies are plentiful, but largely limited to cell cultures or to laboratory animals. Understandably, there are no controlled trials of any form of fasting that gauge the effects on [any cause] mortality [34]. Physicians should be aware that misinformation is pervasive and that their patients often adopt dietary regimens that are far from being clinically validated. Moreover, doctors are often unaware of their patients’ religious or traditional fasting (Table 2) and of its potential health effects.

Based on the evidence we reviewed in this article, no long-term fasting should be undertaken without medical supervision until future research will hopefully help shed further light on fasting and its effects on human health.

## Figures and Tables

**Table 1:** Most common types of fasting.

WEEKLY PLANNING	Day 1	Day 2	Day 3	Day 4	Day 5	Day 6	Day 7
Alternate day fasting, e.g., the “Fast diet”	Ad libitum	0–25% of habitual calorie intake	Ad libitum	0–25% of habitual calorie intake	Ad libitum	0–25% of habitual calorie intake	Ad libitum
Time-restricted feeding/Intermittent fasting	Fast for 16–20 h, then eat within 4–8 h	Fast for 16–20 h, then eat within 4–8 h	Fast for 16–20 h, then eat within 4–8 h	Fast for 16–20 h, then eat within 4–8 h	Fast for 16–20 h, then eat within 4–8 h	Fast for 16–20 h, then eat within 4–8 h	Fast for 16–20 h, then eat within 4–8 h
Whole-day fasts, e.g., for religious purposes	Ad libitum	Ad libitum	Ad libitum	Ad libitum or 24-h fast	Ad libitum	Ad libitum	24-h fast

Modified from [4]. Non-caloric fluid intake is ad-libitum.

**Table 2:** Summary of the different fasting practices in major world religions.

Religion	Form of Fasting
Islam	It is obligatory for Muslims to fast the month of Ramadan (30–31 days) which consists of no food or drink from dawn to sunset. Muslims also commonly fast the first 10 days of the Islamic lunar month Dhul-Hijjah and some Muslims commonly fast the Monday and Thursday of each week and/or the middle 3 days of each fast.
Christianity	Catholic Christians abstain from eating meat, but not fish, on Fridays in the 6-week period before Easter, called Lent. Many Catholics also only eat one full meal a day on the days of Ash Wednesday, the first day of Lent, and Good Friday. Some Protestants observe Lent by abstaining from certain favorite foods or habits such as smoking. A similar method of fasting is the ‘Daniel fast’ which lasts 21 days. The Eastern Orthodox church has different fasting periods, including Lent as well as the Nativity fast, Apostles’ Fast and Dormition Fast. These are often several weeks long and entail fasting from specific food items such as red meat and poultry and sometimes fish, oil, and wine. None of the major denominations in Christianity prohibit taking medications while fasting.
Judaism	There are several days of fasting in Judaism. These include Yom Kippur, Tisha B’Av, the Fast of Gedaliah, the Tenth of Tevet, the Seventeenth of Tammuz and the Fast of Esther. These are single days of fasting from all forms of eating and drinking during this period—with the exception of Yom Kippur and Tisha B’Av where Jews abstained from all oral intake (including water) for 24 h (from sunset to sunset). Historically, reform Jews only observed the Yom Kippur fast while Orthodox Jews the above-mentioned fasts. According to Jewish Law, important and/or regular medications can be taken with drink, and if necessary, with food as well but a patient’s medications should be reviewed by a healthcare professional to ensure the fast as compliant as possible.
Hinduism	Fasting takes many forms from abstaining from meat to only drinking water and milk. The most common fast in Hinduism is Ekadasi, which takes place twice a month and often consists of eating only fruits, vegetables, and milk products (although a small minority abstain from all eating and drinking for 24 h). Many Hindus also fast during the month of Shravan. Hindus are permitted to take medications while fasting.
Buddhism	Lay Buddhists fast by abstaining from meat and certain types of food such as processed foods, two or more times per month. Some Buddhists stop eating after midday every day and some monks go further by abstaining from food for 18 days, drinking only a small portion of water.
Sikhism	Sikhism does not promote fasting except for medical reasons.
Baha’i	Fasting is observed from sunrise to sunset during the Baha’I month of ‘Ala with the complete abstention of food and drink. Patients are permitted to take medications while fasting.

Religious practice is heterogeneous. While certain fasting practices are mentioned, patients may not practice them or may practice them in a manner dissimilar to that described above. Modified from [17].

## Data Availability

Not applicable.
